# Hypersalinity drives physiological and morphological changes in *Limia perugiae* (Poeciliidae)

**DOI:** 10.1242/bio.017277

**Published:** 2016-07-11

**Authors:** Pablo F. Weaver, Oscar Tello, Jonathan Krieger, Arlen Marmolejo, Kathleen F. Weaver, Jerome V. Garcia, Alexander Cruz

**Affiliations:** 1 Department of Biology, University of La Verne, 1950 3rd St., La Verne, CA 91750, USA; 2 Herbarium, Library, Art & Archives Directorate, Royal Botanic Gardens, Kew, Richmond, Surrey TW9 3AE, UK; 3 Instituto de Investigaciones Botánicas y Zoológicas Prof. Rafael M. Moscoso, Universidad Autónoma de Santo Domingo, Santo Domingo, Dominican Republic; 4 Department of Ecology and Evolutionary Biology, University of Colorado, Boulder, CO 80309-0334, USA

**Keywords:** Poeciliidae, Hypersalinity, Osmoregulation, Mitochondria, Geometric morphometrics

## Abstract

A fundamental question in biology is how an organism's morphology and physiology are shaped by its environment. Here, we evaluate the effects of a hypersaline environment on the morphology and physiology of a population of livebearing fish in the genus *Limia* (Poeciliidae). We sampled from two populations of *Limia perugiae* (one freshwater and one hypersaline) in the southwest Dominican Republic. We evaluated relative abundance of osmoregulatory proteins using western blot analyses and used a geometric morphometric approach to evaluate fine-scale changes to size and shape. Our data show that gill tissue isolated from hypersaline fish contained approximately two and a half times higher expression of Na^+^/K^+^ ATPase proteins. We also show evidence for mitochondrial changes within the gills, with eight times more complex I and four times higher expression of ATP synthase within the gill tissue from the hypersaline population. The energetic consequences to *Limia* living in saline and hypersaline environments may be a driver for phenotypic diversity, reducing the overall body size and changing the relative size and shape of the head, as well as impeding the growth of secondary sex features among the males.

## Abstract (precis)

**Summary:** A population of *Limia perugiae* living in hypersalinity shows modifications to osmoregulatory mechanisms, correlated with changes to body size, morphology of the head, and secondary sex features.

## INTRODUCTION

Livebearing freshwater fishes in the genus *Limia* (Poeciliidae) are diverse in their morphology, behavior, and ecology. In terms of overall species diversity and distribution, the genus *Limia* is one of the dominant freshwater fish groups in the West Indies, with upwards of 17 described endemic species occurring on Hispaniola and one endemic species on Cuba, Grand Cayman, and Jamaica (Rauchenberger, 1988; Burgess and Franz, 1989; Rodriguez, 1997; Hamilton, 2001). The broad ecological tolerances and adaptability to varied environments exhibited by these species are likely key to both their colonization and diversification in the West Indies.

Hispaniola, the center of biodiversity for *Limia*, provides a natural laboratory to examine the roles of environmental change in driving divergent evolution. While second in land area to Cuba, Hispaniola is considered to be more topographically diverse, having both the highest point in the Caribbean (Pico Duarte, 3117 m above sea level) and the lowest point (near Lago Enriquillo, 45 m below sea level). *Limia* species exist on all corners of the island, occupying a diversity of aquatic habitats, from cool mountain streams to warm coastal lagoons. The largest assemblage of *Limia* species on Hispaniola is distributed mainly in the southwest Cul de Sac and Valle de Neiba region near Lago Enriquillo in the Dominican Republic. Across this region, *Limia* species exhibit their adaptability by their presence in both freshwater, as well as saline habitats. Several populations even occupy hypersaline environments in the region (Burgess and Franz, 1989).

Here we investigated the role of salinity in driving physiological and morphological change in *Limia*. We focused on the species *L. perugiae* Evermann and Clark, 1906, which is one of the most successful and widely distributed species in the *Limia* genus. Populations of *L. perugiae* occupy freshwater ecosystems, as well as saline [∼35 parts per thousand (ppt)] and hypersaline (40-100 ppt) lakes and coastal lagoons. In the southwestern region of the Dominican Republic, three hypersaline areas are occupied by *L. perugiae*: Lago Enriquillo, Laguna de Oviedo, and the Las Salinas/Las Calderas area ∼100 km SW of Santo Domingo. All three habitats fluctuate in their salinity (ranging from 40-100 ppm) but are by nature consistently hypersaline (Buck et al., 2005). In all three hypersaline localities, *L. perugiae* individuals are known to be smaller and less ornate than their neighboring freshwater populations (P.F.W., unpublished). The goals for this work were to evaluate the role of hypersalinity in driving phenotypic diversity. Specifically, we were interested in identifying the changes to mitochondrial energy production and trans-membrane ion transport mechanisms in the gills and how these may relate to larger morphological changes observed in the field.

Studies show that fish growth and development are dictated by tradeoffs in energy demand and are directly fueled by energy surplus (Ricker, 1975; Chen et al., 1992; Lester et al*.*, 2004). Energy obtained from food is primarily divided into the processes of metabolism, excretion, ion/osmoregulation, and primary reproductive features. The difference between the energy input and the primary allocations of that energy is considered surplus energy, which can then be applied to growth and the development of secondary reproductive features. Extensive research has attempted to quantify and predict the growth of fish, based on changes in energy allocation caused by various changes in the environment, such as temperature, water chemistry, and feed (e.g. Von Bertalanffy, 1957; Lester et al*.*, 2004). The amount of energy that may be allocated for growth can be depicted by the following theoretical equation, adapted from Lester et al*.* (2004):
(1)
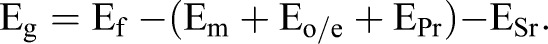
In this equation, energy for growth (E_g_) is dependent on the difference between the energy obtained from food (E_f_) and the essential energy requirements, the minimal energy requirements from metabolism (those needed for the organism to survive; E_m_), osmoregulation/excretion (E_o/e_) and primary reproduction (E_Pr_), and the energy needed for development of secondary reproductive features (E_Sr_).

Under this growth model, any change to the energy demands of survival decreases the amount of energy that may be allocated for growth. Studies have demonstrated that changes in environmental factors, such as water quality and temperature can elicit a decrease in size as a result of the allocation of energy towards processes involved with survival in that environment (Lema and Nevitt, 2006; Horppila et al*.*, 2011; Muñoz et al*.*, 2012). The mechanism for somatic growth is so energy-sensitive that, even at optimal conditions, reproductive needs associated with maturation inhibits further growth. Lester et al*.* (2004) found that during the pre-maturity stage of development, surplus energy is more likely to be allocated towards growth than during the sexually mature adult stage. Reproductive costs (production of spermatozoa or eggs and mate searching) deplete the surplus energy store, leaving less energy for growth. Thus, any deviation in energy expenditure may influence fish morphology. Drastic changes in environment, such as colonizing a hypersaline lagoon, have the potential to create substantial deficiencies of energy.

Osmoregulation in fish is complex and potentially energetically expensive. Previous studies estimate energetic costs at anywhere from 1% to 50% of the energy budget, depending on the tonicity of the environment (Rao, 1968; Nordlie and Lefler, 1975; Nordlie, 1978; Furspan et al., 1984; Nordlie et al*.*, 1991; Toepfer and Barton, 1992; Evans, 2008; Ern et al., 2014). The primary modulators of ions in fish gills are the mitochondria-rich cells, which are the site of the transmembrane ion transport proteins, specifically sodium potassium ATPase (Na^+^/K^+^ ATPase) (Evans et al*.*, 2005). Na^+^/K^+^ ATPase proteins use energy in the form of adenosine triphosphate (ATP) to move sodium ions against their concentration gradient. Previous work has demonstrated substantial changes to the osmoregulatory mechanisms of fish in hypersalinity, at the molecular level, tissue morphology, and organismal physiology (Stuenkel and Hillyard, 1980; Sardella et al., 2004; Bystriansky et al*.*, 2007). For example, one of the primary predictable changes that occurs in most euryhaline teleost fishes is an increase in branchial Na^+^/K^+^ ATPase activity as a way of maintaining osmoregularity in an increasingly saline environment (reviewed by Sakamoto et al., 2001; Marshall, 2002).

Given the energetic demands of osmoregulation and the need for surplus energy for normal growth and development, we can predict that hypersaline environments provide a strong selective pressure on their inhabitants. The colonization of hypersaline waters by *L. perugiae* offers an opportunity to examine morphological changes, as well as physiological responses to an extreme environment and to evaluate populations potentially undergoing divergent natural selection. For fish in hypersalinity, we predicted increased energy demands would lead to changes to the osmoregulatory mechanisms, and that morphological changes would reflect both physiological and environmental differences.

## RESULTS

**Fig. 1.**
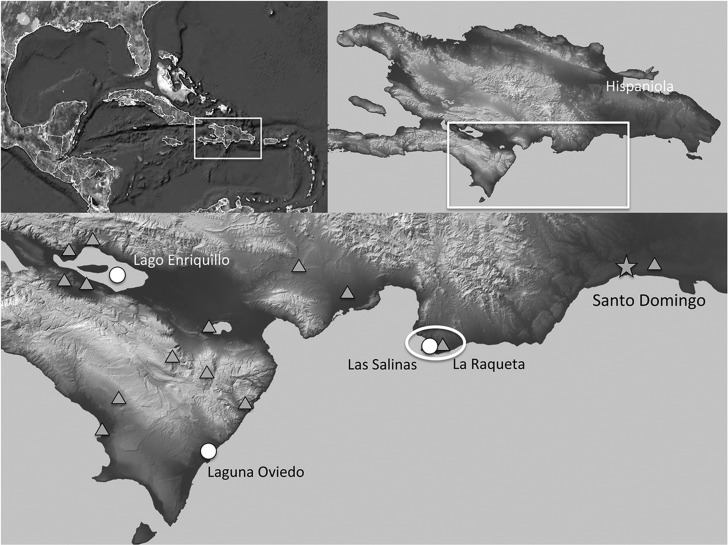
**Map of localities for populations of *L. perugiae* in the southwest Dominican Republic.** Hypersaline populations are indicated by open circles and nearby freshwater populations are represented by shaded triangles. The two populations used in this study are indicated by the oval.

**Fig. 2.**
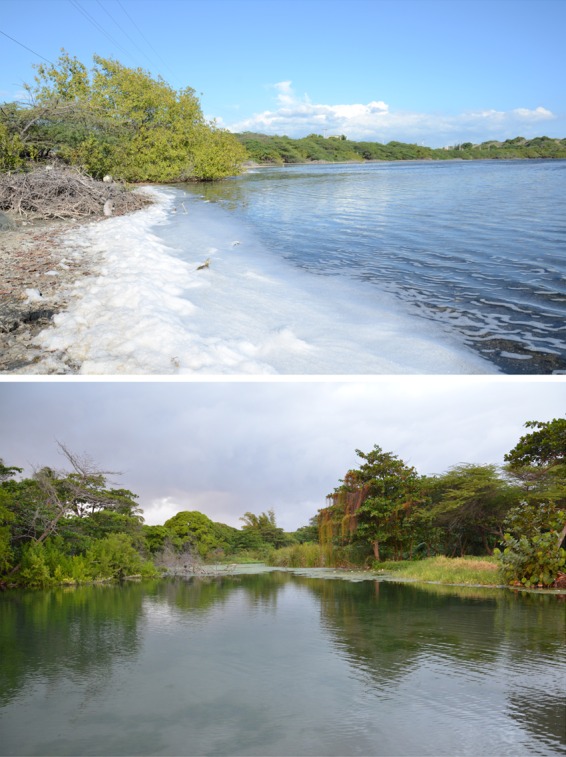
**Habitat photos for the two populations of fish used in this study.** The hypersaline Las Calderas lagoon at Las Salinas is top and the freshwater lagoon at La Raqueta is bottom. Note the salt build-up along the shore of the Las Salinas site.

The water conditions from the hypersaline and freshwater localities (Figs 1 and 2) differed in all environmental measurements, although they were most different in salinity, with the Las Salinas (hypersaline) water measuring 42.08 ppt compared with 0.33 ppt for the La Raqueta (freshwater) site (Table 1). Sites also differed in their dissolved oxygen, with 6.09 mg/l at Las Salinas and 8.12 mg/l at La Raqueta.

**Table 1.**
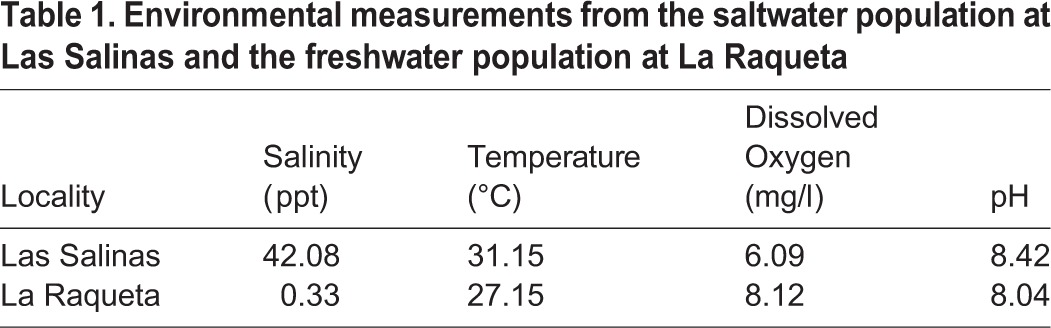
**Environmental measurements from the saltwater population at Las Salinas and the freshwater population at La Raqueta**

### Physiology

**Fig. 3.**
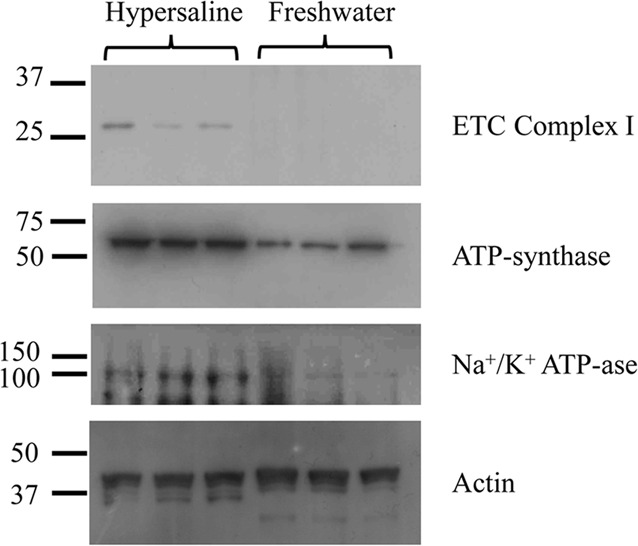
**Gel results from western blot analyses of proteins isolated from the gills of fish found in a hypersaline and a freshwater environment.** For mitochondrial Complex I, 150 mg of protein were resolved on SDS PAGE and treated with monoclonal anti-NDUFS3 (ETC Complex I). For ATP synthase, 150 mg of protein were resolved on SDS PAGE and treated with monoclonal anti-ATP1A (ATP-synthase). For Na^+^/K^+^ ATPase, 150 mg of protein were resolved on SDS PAGE and treated with polyclonal anti-Na^+^/K^+^ ATPase α1 (Na^+^/K^+^ ATPase protein). Gels were run in triplicate to test for consistency and analyzed using densitometry. Gill proteins isolated from hypersaline fish contained approximately eight times more Complex I (band at 30 kDa), four times higher expression of ATP synthase (band at 60 kDa), and two and a half times increase in expression of Na^+^/K^+^ ATPase proteins (band at 112 kDa). Equal protein loading was determined by probing with a monoclonal mouse anti-beta actin antibody (band at 45 kDa).

For this study, mitochondrial and ion transport proteins were analyzed from hypersaline and freshwater fish gills (Fig. 3). Densitometry results showed that fish gills isolated from hypersaline individuals contained eight times more mitochondrial complex I protein, represented by a band at ∼30 kDa, a fourfold higher expression of ATP synthase, represented by a band at ∼60 kDa and finally a two­-and-a-half-fold increase in expression of Na^+^/K^+^ ATPase proteins, represented by a band at ∼100 kDa. All three proteins probed are made up of many subunits, and the antibodies used recognize specific subunits of the protein. The Complex I antibody recognizes the anti-NDUFS3 the iron-sulfur subunit that has a predicted band size of 30 kDa, the ATP synthase antibody recognizes the alpha subunit (anti-ATP5A), which has a predicted band size of 60 kDa, and Na^+^/K^+^ ATPase antibody recognizes the anti-Na^+^/K^+^ ATPase α1 subunit that has a predicted band size of 100 kDa. Bands that depict the specific subunits, when compared to the molecular weight markers, have comparable weights to the predicted band size. Beta actin was used as a loading control and represented by a band at 45 kDa. Although the data show that hypersaline fish have increasing concentrations of Complex I, ATP synthase, and Na^+^/K^+^ ATPase proteins, as determined by the density of the bands, it cannot be determined whether or not this increase in concentration is due to an increase in protein synthesis, from either an increase in mitochondrial content or matrix restructuring of the pre-existing mitochondria, or to a decrease in protein degradation.

### Geometric morphometrics

**Fig. 4.**
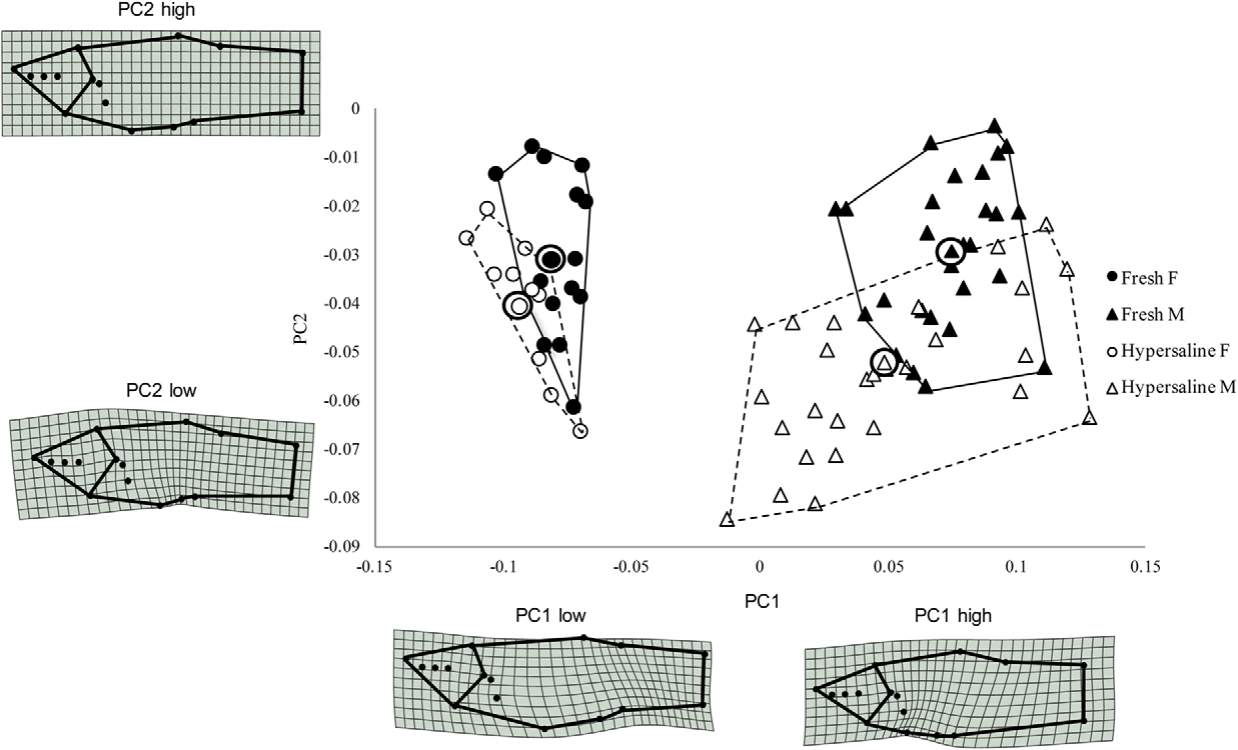
**Scatter plot of relative warp scores along the first two principal component axes.** The hypersaline population from Las Salinas is indicated by open shapes, and the freshwater population from La Raqueta is indicated by closed shapes. Males are indicated by triangles, females by circles. The individuals most closely representing the means are circled. Shape differences can be seen between sexes, as well as between habitat type.

**Fig. 5.**
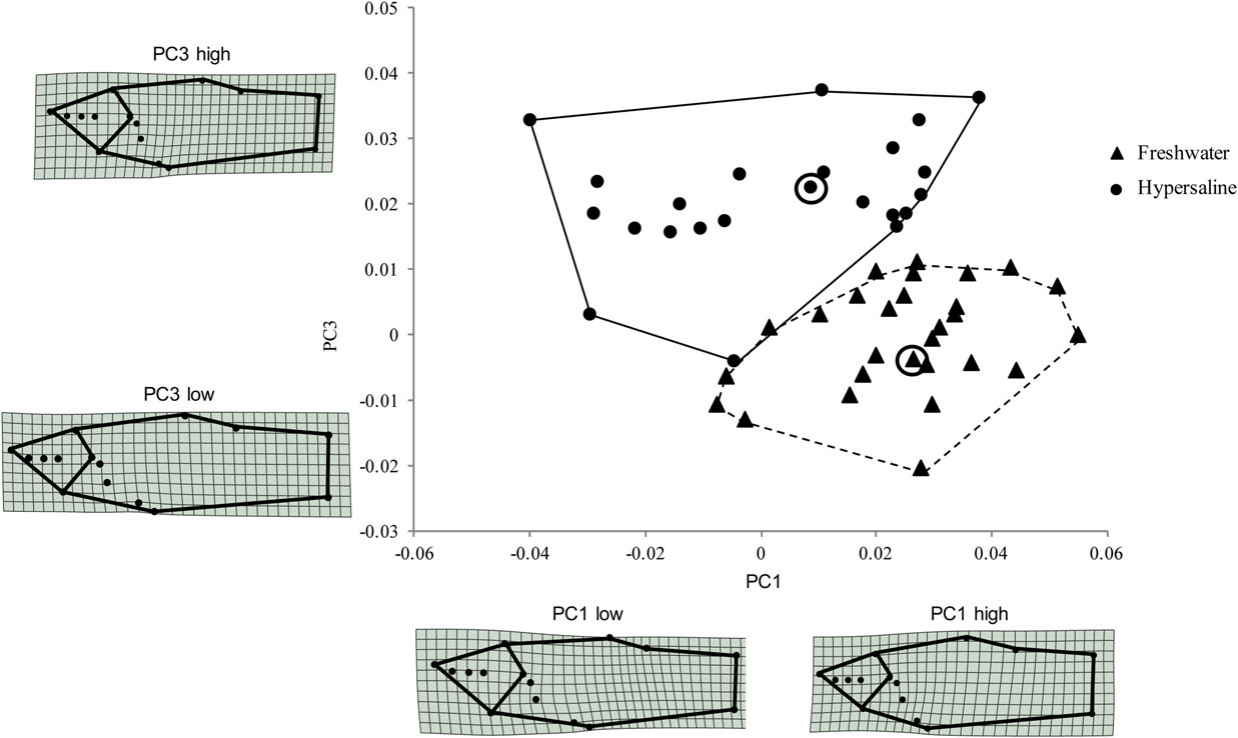
**Scatter plot of relative warp scores along the two most significant principal component axes (PC1 and PC3) from the MANOVA on the male only dataset.** The hypersaline population is indicated by circles and the freshwater population by triangles. Deformation grids showing the specific changes in shape along each axis are shown. Individuals from both populations that are closest to the mean are circled.

In total, we examined 86 individuals from the two sites for the geometric morphometric analysis. Freshwater fish from La Raqueta averaged 3.01±0.79 cm (mean±s.d.) in length and 1.02±0.23 g in mass, and fish from Las Salinas averaged 2.30±0.51 cm in length and 0.27±0.05 g in mass. A plot of the first two principal component axes from the combined dataset of landmark coordinates, including males and females shows that individuals collected in the hypersaline habitat are a distinct ecotype and occupy a different area in morphospace, with lesser scores along PC2, and especially along PC1 (Fig. 4). Differences between populations are more drastic for males than females, but vary in the same way (the means diverge along the same angle with respect to PC1 and PC2). Additional analyses of the male only dataset allowed us to further examine specific aspects of shape that varied between the hypersaline and freshwater ecotypes. In this case, differences between populations can be best observed by PC1 and PC3, with the individuals collected in the hypersaline habitat clumping with lesser scores along PC1 and higher scores along PC3 (Fig. 5).

**Fig. 6.**
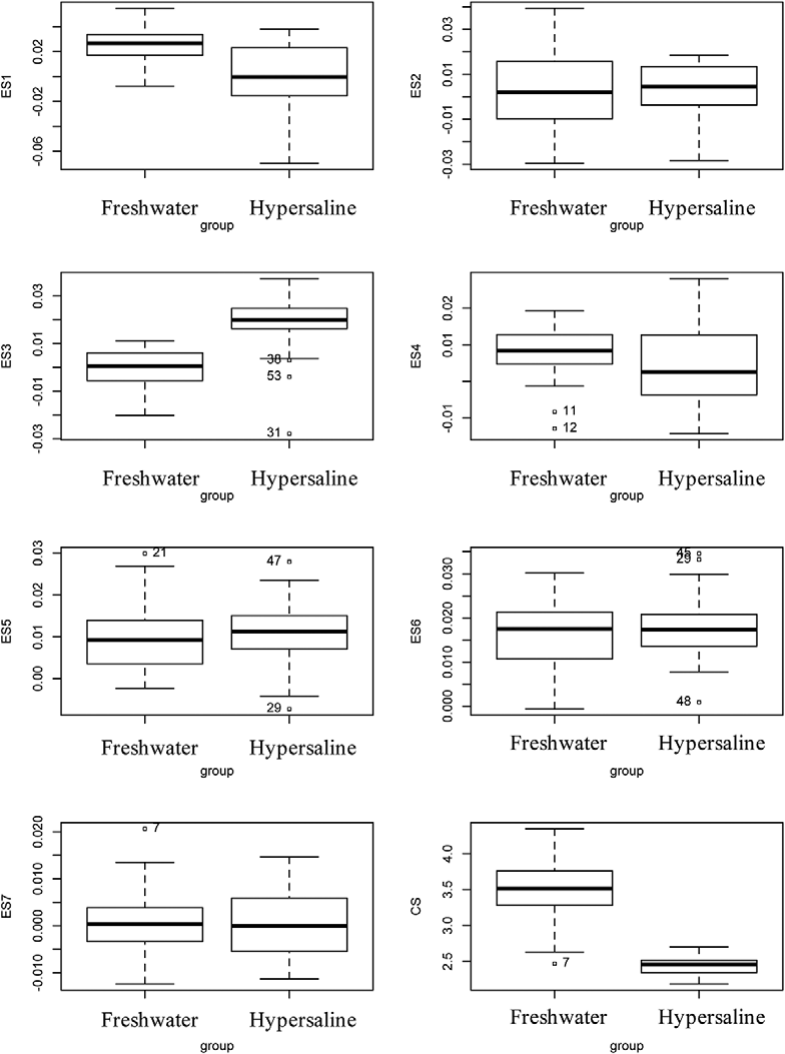
**Morphometric differences between the freshwater and hypersaline ecotypes.** Values are from the outputs box plots show the differences along the seven principle component axes. Difference in centroid size (CS) is also shown.

We ran post hoc tests using GLHT, holding the centroid size constant. Results show that the hypersaline males vary significantly from the freshwater males along ES3 (*P*<0.025), ES5 (*P*<0.025), and ES7 (*P*<0.025) (Fig. 6, Table 2). The results from the MANOVA were slightly different, with significant differences between populations along ES1 (*P*<0.001) and ES3 (*P*<0.001) (Table 3).

**Table 2.**
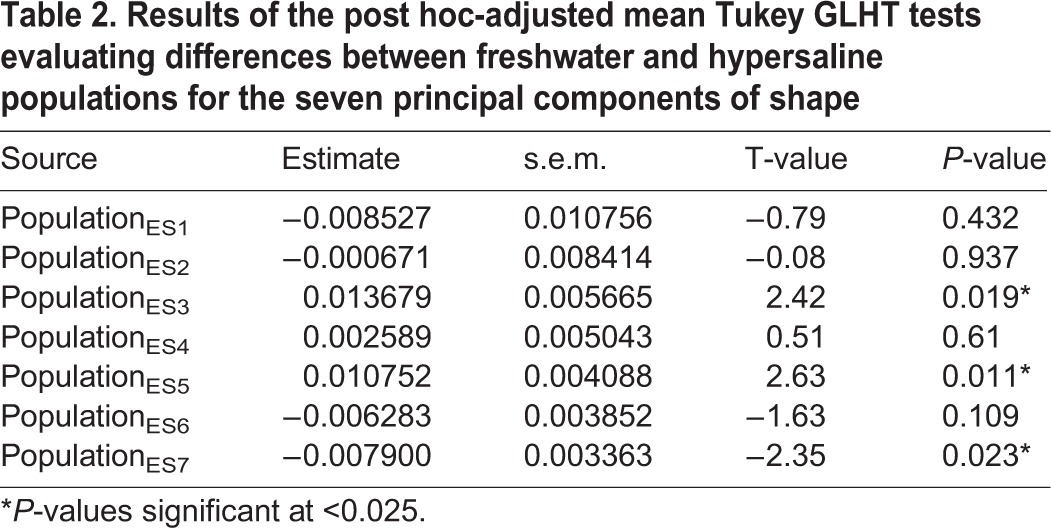
**Results of the post hoc-adjusted mean Tukey GLHT tests evaluating differences between freshwater and hypersaline populations for the seven principal components of shape**

**Table 3.**
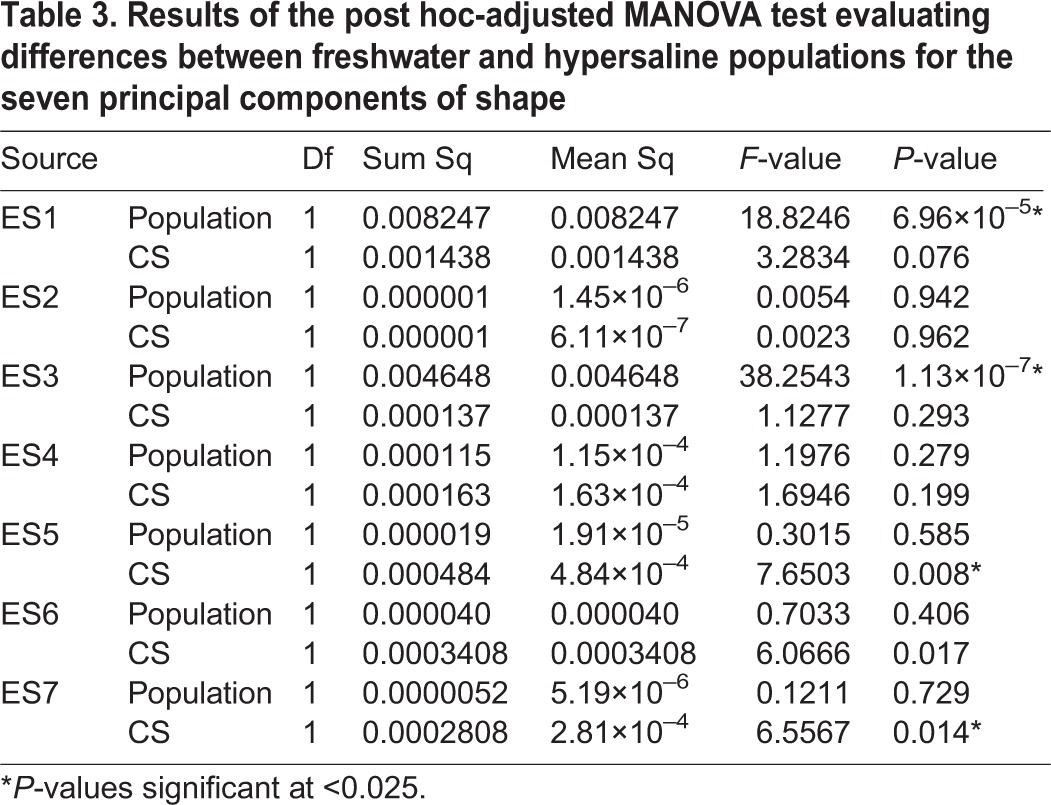
**Results of the post hoc-adjusted MANOVA test evaluating differences between freshwater and hypersaline populations for the seven principal components of shape**

**Fig. 7.**
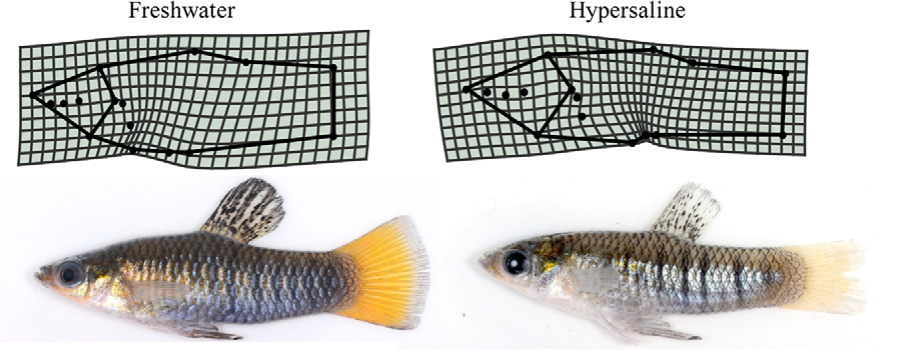
**Comparison of male shape, using mean individuals of each ecotype.** When controlled for size, differences between populations are seen in the head, which is proportionately larger in the hypersaline population, as well as the body shape, which is truncated and more slender in hypersaline males in comparison to the freshwater males.

A comparison of thin plate spline transformation grids allowed us to visualize the shape differences along the principal component axes (Fig. 7). Fish inhabiting the hypersaline habitat differ from the freshwater habitat in the positioning of the fins and in overall head size (proportionately larger for hypersaline), as well as body depth (narrower in hypersaline).

## DISCUSSION

The goal of this work was to evaluate the role of hypersalinity in driving physiological and phenotypic changes in a hypersaline population of the livebearing fish, *Limia perugiae*, from Las Salinas in the southwest Dominican Republic. Given the extreme osmotic challenges presented by hypersalinity, we had two *a priori* predictions regarding the physiological and morphological differences between hypersaline and freshwater populations. First, we predicted changes to the proteins involved in osmoregulation, including mitochondrial and active ion transport proteins. Second, we anticipated a correlation between environmental differences and morphological changes.

### Physiology

As predicted, we observed an upregulation of the active ion transport protein Na^+^/K^+^ ATPase in the hypersaline population. Na^+^/K^+^ ATPase has been shown to be the key transport mechanism for sodium ions across the membrane and is observed to be upregulated in euryhaline fishes acclimating to progressively saltier waters (Langdon and Thorpe, 1984; Evans et al., 2005; Seo et al., 2009). The driving source of energy for active ion transport, as with all cellular processes, is the mitochondria. Energy demands caused by environmental change, such as temperature and salinity, could in concept trigger mitochondrial remodeling, an increase in mitochondria per cell, or an increase in the number of chloride cells in order to accommodate increased bioenergetics needs. The main source of ATP production in the mitochondria is the electron transport system. We focused on complex I because it can be a large contributor to total flux through the electron transport system. Therefore, Complex I has a large influence on the chemo-osmotic potential necessary to drive the ATP synthase complex. Here we show an eightfold increase in Complex I in the gill tissues of hypersaline fish. We also show a fourfold increase in ATP synthase, used to catalyze the production of ATP from ADP. Both processes can be seen as critical mechanisms involved in adjusting to the increased demands of osmoregulation. As Na^+^/K^+^ ATPase proteins are upregulated and are involved directly in the active transport of sodium across the membranes, Complex I and ATP synthase provide the additional fuel necessary to complete the process. In relation to our original growth equation (Eqn 1), our physiology results indicate increased energy allocation towards metabolism (E_m_) and osmoregulation (E_o/e_), with subsequent predicted effects on overall growth, reproduction, and development of secondary reproductive features.

### Morphology

A fundamental question in evolutionary biology is how an organism's phenotype changes in response to its environment. A growing area of research evaluates populations undergoing divergent natural selection to identify the drivers of phenotypic diversity, which are key to the process of ecological speciation (Schluter, 2001; Funk et al., 2002, 2006; Rundle and Nosil, 2005). Studies of divergent natural selection among fishes have mainly focused on biotic interactions (e.g. Endler, 1986; Langerhans and DeWitt, 2004; Langerhans, 2009) and how predator regimes influence features such as coloration, body shape, performance, and life history characteristics. Research has also begun to evaluate the roles of abiotic selective pressures, such as temperature (Lema and Nevitt, 2006; Muñoz et al., 2012; Nilsson et al., 2009), toxins (Riesch et al., 2010; Tobler et al., 2011), and salinity (Boeuf and Payan, 2001; Horppila et al., 2011) in driving population level differences in the morphology and physiology of fishes. Extreme environments should amplify the effects of divergent natural selection, as seen with livebearing fishes in the toxic sulfur pools of Mexico (Tobler et al., 2011).

Our results indicate that the divergent natural selective pressures of the two environments, may be driving phenotypic diversification in *L. perugiae*, including body size, mass, and shape change. In addition to differences in salinity, it is evident that the more saline habitat is also warmer (31°C vs 27°C). If one assumes Q10 values of 2-3 for biological rate functions, this could result in a 30%-60% greater metabolic demand irrespective of salinity (Lema and Nevitt, 2006). The combination of temperature and salinity is likely a powerful selective force in this environment.

Due to the sensitivity of a fish's overall energy balance, any adaptations to osmoregulatory mechanisms or other energetic demands may be diverting energy away from growth and development. Although an increase in mitochondrial respiration in the gills of hypersaline fish may accommodate this extra demand, this only underscores the shifting of energy resources towards the gill area (to feed active transport) and away from other bodily needs. Overall, the morphological feature to change the most was body size, with fish from the hypersaline population measuring roughly three quarters the size and mass of the freshwater counterparts. Another observable difference between populations was the relative size of the head, with the hypersaline fish exhibiting proportionally larger heads than the freshwater fish (Figs 4, 5 and 7). Similar patterns have been seen in fishes inhabiting toxic sulfidic environments (Tobler et al., 2011). As in the sulfidic environment study system, the larger head and gill space may be an adaptation to increased respiratory demands of an extreme environment.

Morphological change can be seen most dramatically with males, who differ most in overall body size, as well as some other interesting shape features. Freshwater males develop distinct secondary sex features, including a broader body and elongated fins, used in courtship display behavior (Farr, 1984; Applebaum and Cruz, 2000; Munger et al*.*, 2004). While we did not include fin shape analysis in this study, differences in overall body shape are evident, with hypersaline males being narrower bodied and more juvenile in appearance. An interesting follow up to this study would be to study population dynamics in the wild as well as to conduct behavioral experiments to evaluate differences in both male breeding behavior and female choice. Behavioral experiments would allow us to evaluate whether sexual selection is a factor in the hypersaline environments, and more importantly, whether reproductive isolating mechanisms, a precondition for ecological speciation (Schluter, 2001; Langerhans, 2009), may be developing in these populations.

An important question, and one that will need to be answered in order to evaluate the true significance of our results, is whether the changes we have seen are truly adaptations in an evolutionary sense, or whether these changes are due to phenotypic plasticity, as seen in the gills of salmonids and eels, and the morphology of three spine sticklebacks as they transition between salinity gradients (Mazzarella et al., 2015). It cannot be ruled out that changes to the ion transport proteins and mitochondrial respiration are highly inducible in fish and that *L. perugiae* can be cross acclimated to an opposite salinity regime (P.F.W., unpublished). This potential underscores the need for further laboratory studies on the differences between a fish's acclimatization response and long term adaptation. Studies on tilapia have shown phenotypic plastic responses in gill morphology under hypoxia, as well as genetic assimilation of those changes over generations (Chapman et al*.*, 2000). Heritable change is also supported through our own evidence for lab-reared generations of hypersaline populations maintaining the dwarfed appearance of their wild-caught ancestors.

In conclusion, the *Limia* of the West Indies are a showcase for evolution and adaptation to novel environments. While poeciliids continue to provide fertile ground for studies of ecological speciation, little work has evaluated the physiological/biochemical changes that underlie the mechanisms for adaptation. *L. perugiae* offers a new system to evaluate some of the potential inner workings of adaptations under divergent natural selection.

## METHODS

### Fish sampling

All experiments were conducted under guidelines and protocols approved by the IACUC committee at the University of La Verne, and fish were sampled and transported with appropriate permits from wildlife agencies in the Dominican Republic. Fish used in this experiment were collected in the wild from the Las Salinas area of the Dominican Republic in December 2013 (Figs 1 and 2). Adult fish of both sexes were randomly sampled from the wild in each of the following areas: ‘hypersaline’ individuals were collected from the Las Calderas lagoon (18.2125 N, 70.5397 W) and ‘freshwater’ individuals were collected near the neighboring town of La Raqueta (18.2311 N, 70.3601 W).

Environmental variables of water temperature, salinity, pH, and dissolved oxygen were measured using a YSI Model 556 multi probe (YSI Inc., Yellow Springs, OH USA). All fish were caught using seine nets and were transferred to 37-liter holding containers of their native waters. Live fish were then transported to the University of La Verne for western blot analysis.

### Western blotting

Previous research has demonstrated that during the process of acclimating to higher salinity, fish significantly increase both the number and activity of Na^+^/K^+^ ATPase proteins, as well as sodium/potassium chloride co-transporters (Scott et al., 2004; Tseng and Hwang, 2008; Tse and Wong, 2011). As the upregulation of the two proteins are coupled, and ultimately dependent on the pumping of Na^+^ outside the cell by Na^+^/K^+^ ATPase, we focused on the Na^+^/K^+^ ATPase protein (alpha 1 subunit) and the changes induced in the biosynthesis of ATP via aerobic metabolism, specifically Complex I (NDUFS3 subunit) and ATP synthase (alpha subunit of Complex V, one of the 18 subunits encoded by the nuclear and mitochondrial genes). Three fish were randomly sampled from each locality (hypersaline and fresh) and were sacrificed with an MS-222 (3-aminobenzoic acid ethyl ester) (Sigma, St. Louis, MO USA) overdose, after De Tolla et al. (1995) and Reed and Jennings (2010). The gills were excised and homogenized in SEI Buffer, containing 0.3 M sucrose, 0.02 M EDTA, 0.1 M imidazole, and 0.1% CHAPS. Proteins were isolated via perchloric acid precipitation, and concentration was determined using a NanoDrop Lite Spectrophotometer (Thermo Scientific, Waltham, MA USA). 150 mg of protein was placed in 1× Pierce Lane Marker Reducing Sample Buffer (#3900; ThermoFisher Scientific, Inc., Grand Island, NY USA), loaded with saltwater fish on one side of the gel and freshwater fish on the other, and resolved in 10% SDS PAGE. The protein was then transferred to a polyvinylidene fluoride (PVDF) membrane, blocked with casein (#161-0782; Bio-Rad, Hercules, CA USA), and probed with Complex I, ATP synthase, and Na^+^/K^+^ ATPase antibodies for two hours at room temperature. Mitochondrial antibodies were obtained from Mitosciences (Eugene, OR USA) against Complex I (anti-NDUFS3; 1:2000; ms110-ab110246 mouse monoclonal antibody) and ATP synthase [anti-ATP5A (the alpha subunit); 1:1000; ms502-ab110273 mouse monoclonal antibody). Anti-Na^+^/K^+^ ATPase α1 subunit antibody was purchased from Santa Cruz Biotechnology, Inc. (Santa Cruz, CA USA) (1:1000; sc-16043 goat polyclonal antibody). Manufacturers' protocols lists zebrafish proteins as cross-reactive with all antibodies. To ensure equal loading, the gel was probed with a monoclonal mouse anti-beta actin antibody (1:1000; ab170325; Abcam, Cambridge, MA USA). The anti-actin antibody was incubated for two hours at room temperature. Membranes were washed and blocked again with casein, then probed with a secondary goat anti-mouse (sc2005) or donkey anti-goat antibody (sc2020; both at 1:2500) secondary antibodies from Santa Cruz Biotechnology, Inc., for one hour at room temperature. Chemiluminescence was captured with Hyblot CL Autoradiography Film (e3012) obtained from Denville Scientific Inc. (Holliston, MA USA) and band intensity compared between samples using Image Studio Lite Software package (version 3.1).

### Geometric morphometrics

**Fig. 8.**
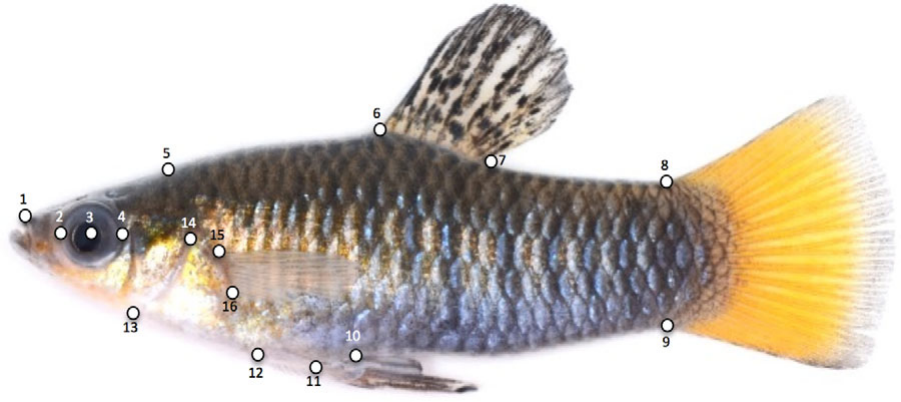
**Sixteen landmark positions used for a landmark-based analysis.** Pictured is a male from the freshwater population of La Raqueta. We digitized the following landmarks: (1) the tip of the upper jaw, (2) the anterior, (3) center, and (4) posterior most edge of the eye, (5) the posteriodorsal tip of the supraoccipital crest, (6) the anterior and (7) posterior insertion of the dorsal fin, (8) the dorsal and (9) ventral insertion of the caudal fin, (10) the posterior and (11) anterior insertion of the anal fin, (12) the anterior junction of the pelvic fins and the ventral midline, (13) the ventral most junction of operculum and the body midline, (14) the posterior most edge of the operculum, (15) dorsal and (16) ventral insertion of the pectoral fin.

Mass and standard length of fish from nose to base of the caudal fin were recorded for all individuals. We used landmark-based geometric morphometrics to quantify phenotypic differences between individuals from the freshwater and hypersaline populations*.* Previously sacrificed fish, whose gills had been excised and used for protein analysis, were immediately prepped for photography. Additional individuals were sacrificed in MS222, so that overall we photographed 29 males and 16 females from the freshwater La Raqueta population and 29 males and 12 females from the saltwater Las Salinas population. Our sampling effort here was as high as possible to increase statistical power, with consideration given to not oversampling from the population. Only mature adults were included in the analysis. High resolution images were taken with a Nikon (Tokyo, Japan) 7000 camera body and a 105 mm Nikkor macro lens, following the protocol of Schutz and Krieger (http://www.morpho-tools.net/softwareguide/GM%20guide%20v4%20OLs.pdf). Sixteen landmarks (Fig. 8) on each image were then digitized using tpsDig (http://life.bio.sunysb.edu/morph/). We chose landmarks that represented reproducible points around the body and the head/opercular region (modified from Tobler et al., 2011). This allowed us to quantify body shape and size, as well as heuristically examine relative head size in relation to body size via deformation plots.

Shape variation was analyzed using Procrustes PCA, mathematically equivalent to performing an unweighted (with respect to bending energy) relative warps analysis (Zelditch et al., 2012). One of the most widely used programs for geometric morphometric analysis of landmark data, tpsRelw (http://life.bio.sunysb.edu/morph/), uses unweighted relative warps by default, so these results will be identical. Procrustes PCA is computationally simpler and more flexible for further statistical analysis of the resulting morphospace. Landmark data was subjected to a Procrustes superposition, which aligns all specimens, removing differences due to size, translation, and rotation. The Procrustes shape coordinates were then subjected to a covariance-based principal components analysis. This yielded a morphospace composed of a series of orthogonal, variance-optimized axes, each describing some aspect of shape variation in the sample. Variation along these axes was modeled using thin plate splines to visualize the nature of shape variation along selected axes (in particular, those that showed separation between populations and/or sexes).

In order to focus on some of the finer scale shape changes, we conducted an additional Procrustes PCA analysis on the male only dataset. Significant differences in shape variables were assessed using several methods in the R statistical package (R Core Team, 2013). For this analysis, we focused specifically on the first seven principal component axes, as they accounted for approximately 90% of the variance. We assessed the data for interaction effects, but determined that interaction between PC1-7 were not significant (data not shown). This allowed us to proceed with a simple linear model with a covariate approach. We assessed the marginal means (means of the groups adjusted for the covariate of centroid size) for the groups using GLHT in multcomp (http://multcomp.r-forge.r-project.org/). We also assessed differences between groups using MANOVA as implemented in R.
